# Creating a Digital Toolkit to Reduce Fatigue and Promote Quality of Life in Multiple Sclerosis: Participatory Design and Usability Study

**DOI:** 10.2196/19230

**Published:** 2021-12-09

**Authors:** Sarah Thomas, Andy Pulman, Huseyin Dogan, Nan Jiang, David Passmore, Keith Pretty, Beth Fairbanks, Angela Davies Smith, Peter W Thomas

**Affiliations:** 1 Bournemouth University Clinical Research Unit Department of Medical Science & Public Health, Faculty of Health & Social Sciences Bournemouth University Bournemouth United Kingdom; 2 Department of Computing & Informatics Faculty of Science & Technology Bournemouth University Bournemouth United Kingdom; 3 Bristol & Avon Multiple Sclerosis Centre Southmead Hospital North Bristol National Health Service Trust Bristol United Kingdom

**Keywords:** multiple sclerosis, fatigue, self-management, cognitive behavioral, digital health, mHealth, eHealth, development, participatory design, usability testing

## Abstract

**Background:**

Fatigue is one of the most common and debilitating symptoms of multiple sclerosis (MS), experienced by more than 80% of people with MS. FACETS (Fatigue: Applying Cognitive Behavioral and Energy Effectiveness Techniques to Lifestyle) is an evidence-based, face-to-face, 6-session group fatigue management program for people with MS. Homework tasks are an integral part of FACETS and are currently undertaken in a paper-based form. Feedback from a consultation undertaken with FACETS attendees and health care professionals with experience in delivering the FACETS program suggested that being able to complete the homework tasks digitally would be desirable, potentially enhancing engagement and adherence and enabling on-the-go access to fit into busy lifestyles. Relative to other long-term conditions, there are few apps specifically for MS and, of those available, many have been developed with little or no input from people with MS.

**Objective:**

The purpose of this mixed methods study was to create a digital toolkit comprising the homework tasks (eg, activity diary, goal planner, thought diary) of the FACETS program for people with MS, considering end users’ unique requirements throughout the design, build, prototyping, and testing stages.

**Methods:**

Phase 1 involved the elicitation of detailed user requirements for the toolkit via 2 focus groups with previous attendees of FACETS (n=3 and n=6) and wireframing. Phase 2 involved supervised usability testing with people with MS (n=11) with iterative prototyping. The usability sessions involved going through test scenarios using the FACETS toolkit on an Android test phone with video capture and concurrent think-aloud followed by completion of the System Usability Scale (SUS) and a semistructured interview collecting feedback about design, content, and functionality.

**Results:**

The mean SUS score for the digital toolkit was 74.3 (SD 16.8, 95% CI 63.2-85.6; range 37.5-95), which equates to an adjective rating of good and a B grade (70th-79th percentile range) on the Sauro-Lewis curved grading scale. A number of usability and design issues (such as simplifying overall screen flow to better meet users’ needs) and suggestions for improvements (such as using location-based services and displaying personalized information and progress via a central dashboard) were addressed and implemented during the usability testing cycle.

**Conclusions:**

This work highlights the importance of the participation of people with MS across the entire development cycle, working to a human-centered design methodology to enable a considered and MS-centered solution to be developed. Continued horizon scanning for emergent technological enhancements will enable us to identify opportunities for further improvements to the FACETS toolkit prior to launch. The toolkit supports self-monitoring and management of fatigue and has potential applicability to other long-term conditions where fatigue is a significant issue.

## Introduction

### Background

Multiple sclerosis (MS) is a neurological condition affecting the central nervous system. More than 2.5 million people worldwide have MS with over 130,000 in the United Kingdom [1,2] at an estimated cost to the UK economy of £3.3 to £4.2 billion (US $4.5 to $5.7 billion) per annum [3]. Fatigue is one of the most common and debilitating symptoms of MS [4], experienced by over 80% of people with MS [5], and the main reason for stopping work early [6]. FACETS (Fatigue: Applying Cognitive Behavioral and Energy Effectiveness Techniques to Lifestyle) is a group-based, face-to-face fatigue management program for people with MS developed by members of our team that has been shown to be effective in a national multicenter randomized controlled trial (RCT) funded by the UK MS Society [7-11]. In response to the COVID-19 pandemic, some health care professionals have been delivering the FACETS program virtually (via video conferencing) with initial participant feedback promising.

The program is delivered in 6 weekly sessions [8]. A key component of the FACETS program is the homework tasks that provide an opportunity for participants to try out what they have learned and put it into practice in their daily lives (Table 1).

**Table 1:** Overview of the FACETS^a^ program.

Session no.	Session title	Homework elements
1	What is MS^b^-related fatigue?	Activity and fatigue diary; energy measure
2	Opening an energy account	Rest, activity, and sleep planner
3	Budgeting energy and smartening up goals	Goal-setting exercise
4	Stress response; cognitive behavioral model	Fatigue thought diary
5	Putting unhelpful thoughts on trial	Thought challenge sheet
6	Recapping and taking the program forward	Keeping-on-track planner

^a^FACETS: Fatigue: Applying Cognitive Behavioral and Energy Effectiveness Techniques to Lifestyle.

^b^MS: multiple sclerosis.

Given the favorable results from the national multicenter RCT of FACETS [9-11] and subsequent rollout of the program, team members conducted a consultation on behalf of the MS Society to gather views from stakeholders (people with MS and health care professionals) about potential digital delivery models to enhance reach [12]. Self-guided web-based delivery models for fatigue management have shown promise in MS, although dropout rates have tended to be relatively high [13,14], a common issue encountered in eHealth trials [15]. Findings from the consultation indicated that stakeholders considered an online delivery model of FACETS to be of value [12], although not a direct replacement for the face-to-face version. An online e-learning package was deemed the best way to deliver a minimum viable solution—a product requiring only a limited amount of development time that is implemented with a minimal number of features to provide a basic working model with scope for future expansion and improvement. This has now been launched by the MS Society [16].

During the consultation, it also became apparent there were no high-quality free apps that could support digital completion of the FACETS homework tasks. A separate key recommendation, therefore, was to initiate a project to create a free stand-alone digital toolkit consolidating the structured homework elements of the FACETS program [12]. This project forms the focus of our paper.

Smartphone ownership and use is high in people with MS [17-19]. An app format would have the advantage of permitting on-the-go access and real-time symptom logging and use of reminders, potentially enhancing adherence to the homework tasks [12,20].

Everywhere I go, I’ve got my phone. If I’ve got a few minutes, I sit and fill it in and if it’s fresh and current, I wouldn’t fill in paperwork. Even on the course, I’d fill in the paperwork the night before, or the morning I was coming to the class. But if I had it on my phone, I’d be more inclined to fill it in.

Previous attendee of the face-to-face FACETS program

12

The 2018 MS Society/Nuffield Trust data and technology report presents a vision of “personalized, coordinated, and empowering care for people with MS, enabled by effective technology,” noting that “Digital transformation and the possibilities it provides have not yet been realised within the care and support people with MS access” [21,22]. A complementary mobile solution enabling the FACETS homework elements to be made interactive and portable aligns closely with recommendations from the MS Society data and technology report and action plan, particularly in the areas of having more control over care and accessible and coordinated care [21-23]. It would help to meet the aims of the UK MS Society research strategy in relation to self-management and implementation [24] and would address the third (fatigue) and fourth (self-management) James Lind Alliance research priorities for MS [25].

In their 2017 systematic review of MS apps available from US app stores, Giunti et al [26] noted there were few apps available for MS relative to other long-term conditions such as cancer and diabetes [27-29]. While there are mobile apps available for fatigue management in MS [30-33], they did not meet our requirements of being free to use. To date, only a fatigue management app for cancer, which draws upon cognitive behavioral principles, has been evaluated in a full-scale RCT [34-36]. While there are separate apps available to support self-management of MS symptoms (including diaries and symptom loggers), these did not align sufficiently with the FACETS homework elements, and most were not free.

### Preliminary Work

Findings from a 2018 systematic search and scoping review by Giunti et al [37] suggested that most MS-specific apps lack features desired by people with MS. They and others have called for greater involvement of people with MS and health care professionals before digital solutions are implemented [37-40], noting the importance of understanding condition-specific factors when designing mobile health (mHealth) apps [37-39]. Developers need to consider the requirements of people with MS and possible MS symptoms (blurry vision, reduced fine motor control, cognitive impairment, and fatigue) throughout the development, prototyping, testing, and implementation of any digital solution [21-23,38,41,42].

Initial requirements for the FACETS toolkit were categorized using the MoSCoW framework [43], a simple method used across business disciplines to enable project teams and stakeholders to define requirements: must have (a necessity for meeting the specified goal), should have (beneficial but not essential for a successful product), could have (desirable but not important), and won’t have *this time* (future possibilities but not feasible for immediate implementation). Findings from the consultation [12] informed the initial set of draft requirements, and a stakeholder workshop and interviews with service users were conducted to explore client expectations regarding digitization and begin gathering baseline requirements [44]. An affinity diagram and personas (fictional characters that incorporate composite attributes of target users) were created and used in the early design phases [44]. A card-sorting task (involves ordering, grouping, and naming of objects or concepts) was undertaken with the research team to guide the design of the navigational structure [44]. Paper designs were sketched leading to the creation of low-fidelity wireframes and, subsequently, to the first interactive high-fidelity design prototype.

The toolkit was primarily aimed at complementing the existing FACETS face-to-face program and the MS Society online course. This meant that it needed to remain tethered to the existing materials and the way they are structured to ensure consistency. A secondary consideration was that there might be users downloading the toolkit who had not attended FACETS but who might wish to use elements of the toolkit for recording data relating to their MS and fatigue. A further consideration was that in the future the toolkit might be used to share information with health care providers [45] or expanded to include more content from the FACETS program; the toolkit might also be considered relevant for other long-term conditions [46].

Choosing the type of technology to use for the toolkit was challenging as this is a constantly evolving area. One recommendation from Beatty et al [47] in the context of an evaluation of an intervention for cancer-related distress was that future online programs should be multiplatform to facilitate access across a full range of devices. The FACETS toolkit was initially developed for Android—chosen due to its larger market share and lower anticipated cost of development compared to Apple iOS [48]. Given that a key requirement was access to the toolkit without Wi-Fi and that it would potentially need to align with the MS Society’s e-learning course [16] (in addition to the face-to-face program) [7], mobile web solutions were not considered suitable. The toolkit was developed using an agile approach following Google’s Material Design guidelines [49] and industrial best practices, with reference to the adapted technology acceptance model [50-52]. Google user interface guidelines [53] were followed when creating and using icons and widgets, and core app quality guidelines [54] were followed to ensure a baseline satisfactory user experience. Coding started in October 2018. Several prototypes were developed that incorporated all MoSCoW requirements identified as must have and should have [43]. Initial versions concentrated on implementing basic operational functionality that could then be expanded upon or altered as required.

## Methods

### Study Design

This was a mixed methods study [55] involving quantitative approaches (MS-specific and demographic questions and the System Usability Scale [SUS] [56]) and qualitative approaches (focus groups, think-aloud protocol, and semistructured interviews). Ethical approval for this research was obtained from Bournemouth University (ref. 17430).

### Participants and Recruitment

**Figure 1:**
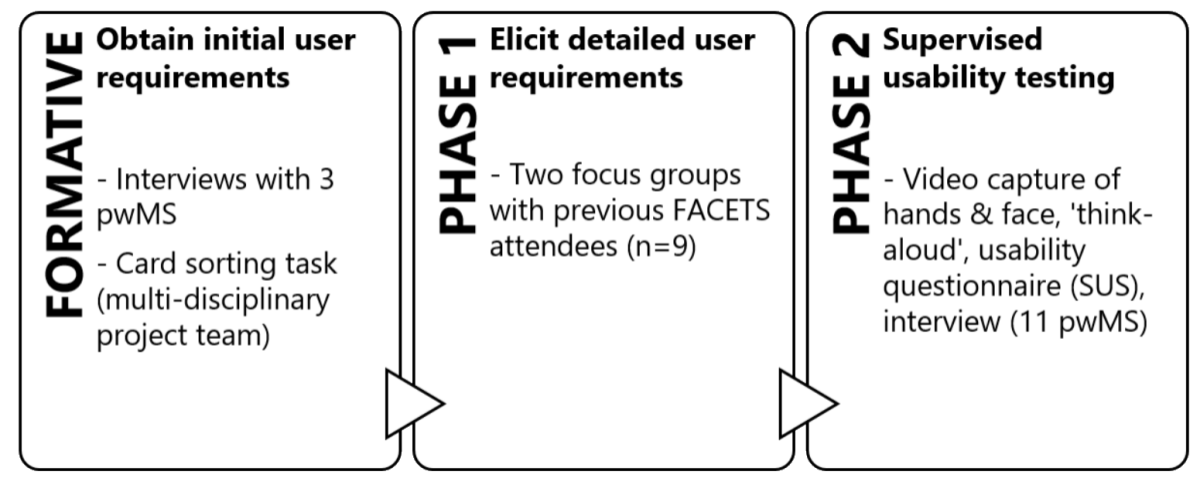
Schematic of study phases. FACETS: Fatigue: Applying Cognitive Behavioral and Energy Effectiveness Techniques to Lifestyle; pwMS: people with multiple sclerosis; SUS: System Usability Scale.

Participants were recruited via a local MS center (individuals who had participated in a previous study and had given permission to be contacted about future research), a local MS support group mailing list, and via an advertisement on the MS Research, Treatment, and Education website. For both the phase 1 focus groups and phase 2 supervised usability testing studies, participants who contacted the research team were emailed participant information sheets with information about the study. Inclusion criteria included being age 18 years or older, having a self-reported diagnosis of MS, experiencing fatigue impacting daily life, and being an active smartphone user (phase 1 only). See Figure 1 for schematic of phases of study.

### Procedures

The focus groups (phase 1) were held at a conference center in Bristol. Supervised usability sessions (phase 2) were held at a conference center in Bristol and on the Bournemouth University campus. Both venues were accessible, and taxi-booking and reimbursement of travel expenses were offered to all participants. To minimize participant burden, we obtained written informed consent via a participant agreement form on the day of the focus groups or usability sessions. In phase 2, we audiorecorded and filmed some parts of the supervised usability testing sessions. If participants did not wish to be audiorecorded or filmed, we offered a one-to-one session with notes taken as an alternative. Two copies of the agreement form were countersigned by the researcher. One copy was given to participants for their records, and one copy was retained by the researcher. The main ethical consideration related to fatigue, which is a major issue for people with MS. Focus groups and usability sessions included regular rest breaks and provision of refreshments, and we emphasized that participants could take a break or stop participating at any time. Duration of sessions was no longer than 90 minutes.

#### Phase 1. Focus Groups to Elicit User Requirements

This phase involved the elicitation of detailed user requirements and wireframing. Two focus groups (n=3 and n=6) with previous attendees of the FACETS face-to-face program were held to gather feedback about requirements and preferences for the toolkit (Multimedia Appendix 1). These were facilitated by AP (in Bristol, an MS assistant practitioner also attended) and audiorecorded and transcribed verbatim. A second set of design prototypes was then created using an interactive prototyping tool, and feedback was obtained from focus group attendees in person and via paper-based semistructured questionnaires completed after attendance. Based on this feedback, a third set of design prototypes was created prior to development commencing.

#### Phase 2. Supervised Usability Testing Sessions

**Figure 2:**
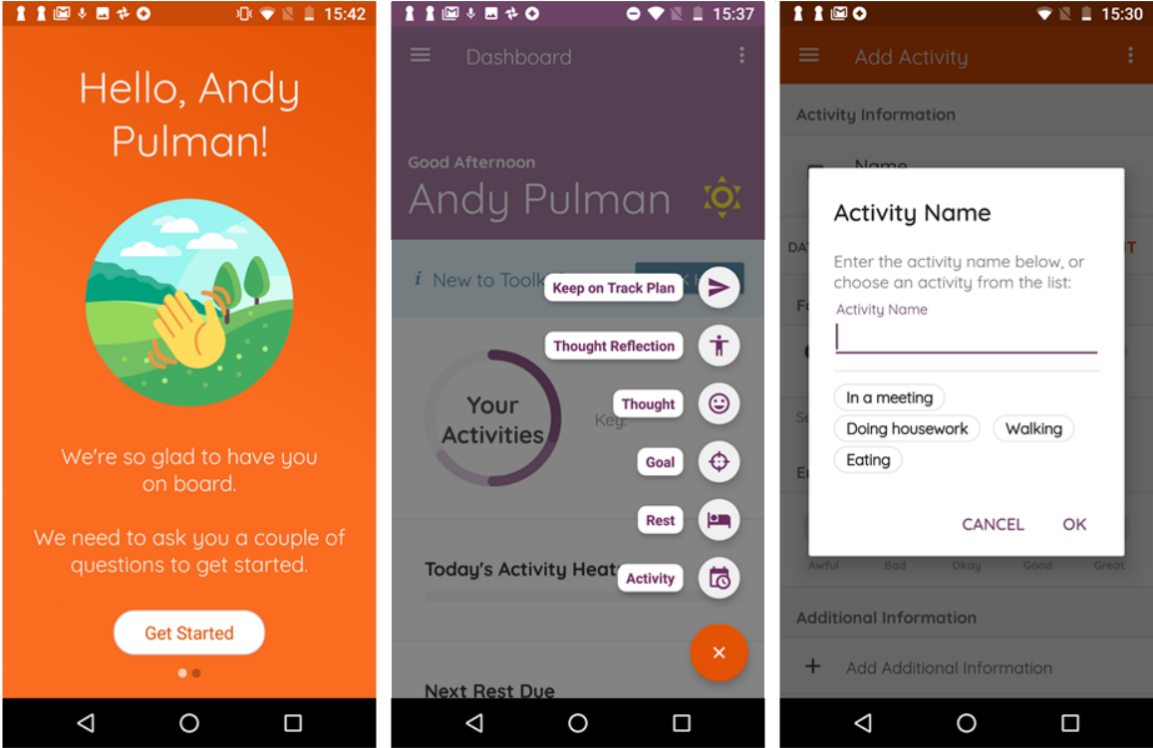
Dashboard and homework element screenshots from working prototype v0.0.3.

Participants (n=11) were asked to use elements of the toolkit and complete up to 2 specific test scenarios (lasting 30 minutes in total) on a supplied Android mobile phone (Table 2). Videocapture of their hands (via a usability rig) and face/top half of their body was undertaken as they interacted with the toolkit while concurrently thinking aloud (give a running commentary). They also completed demographic and MS-specific questions and the SUS [56]. Participants were asked about initial impressions of the version of the toolkit they had tested and for feedback on its design, content, and functionality during a subsequent 30-minute (audiorecorded) semistructured interview based on a topic guide (Multimedia Appendix 2). The digital health postdoctoral researcher (AP) led the usability sessions (n=11). The developer (DP) attended all sessions, and ST (research psychologist) attended 9 sessions. Testing commenced with a stable prototype release; later release versions were created based on user feedback and tested with users iteratively (see Figure 2 for example screenshots of a prototype release).

**Table 2:** Mapping of FACETS^a^ sessions to the toolkit elements and usability test scenarios.

Homework task in face-to-face program	Toolkit element	Test scenarios	Participants (1-11) who tested element
Week 1: activity and fatigue diary; energy measure	Activity diary	Add one or more activities (eg, vacuuming, swimming, making breakfast, gardening) performed recently. Make a change to the activity just added, and view it to check that the information was amended correctly.	1-5
Week 2: rest, activity, and sleep planner	Rest and sleep routine	Thinking about your wake-sleep routine, add a wind-down alarm (for when typically planning to start getting ready for bed) and a wind-up alarm (for when typically planning to start to wake up in the morning). Then create one or more rest periods during the day, setting the duration (scheduling for times when you might wish to take a rest during a typical day). Test out a few alarm options and see if they work correctly.	1-3
Week 3: goal-setting exercise	Goal planner	Create one or more SMART (specific, measurable, achievable, realistic, time for review) goals (a lifestyle change you would like to make). Some possible ideas for areas for change could be exercise routines, relaxation practice, incorporating rest periods, and establishing sleep-wake routines.	4-9
Week 4: fatigue thought diary	Thought diary	Think about a situation that triggered strong emotions and unhelpful thoughts related to fatigue. Describe the situation in the thought diary along with up to 3 unhelpful thoughts, and select the emotions experienced at the time. Then rate strength of belief for each unhelpful thought and for the accompanying emotions.	10-11
Week 5: thought challenge sheet	Thought diary	Select a thought to challenge. Start off by identifying any unhelpful thinking styles, think of one or more alternative thoughts, and rate strength of belief. Then rerate strength of belief in the original thought, and rerate the strength of the associated emotions.	10-11
Week 6: keeping-on-track planner	Keeping-on-track planner	Help the user maintain momentum: complete a plan that focuses on the next 3 months (this element is still under development).	—^b^ (element not yet developed)

^a^FACETS: Fatigue: Applying Cognitive Behavioral and Energy Effectiveness Techniques to Lifestyle.

^b^Not applicable.

### Analysis

The SUS [56] is a standardized questionnaire for collecting usability evaluations of a system being tested and has been shown to have good validity and reliability [57], including in the evaluation of mobile health apps [58]. Standard scoring is between 0 and 100 [57], which the Sauro-Lewis curved grading scale [59] converts to a normative percentile score and associated grade. These grades can range from A (best imaginable on the adjective rating scale by Bangor et al [60]) to F (worst imaginable). Participant ratings on the SUS were collected during 2019. Quantitative ratings from the questionnaire were summarized using descriptive statistics.

Focus group and interview recordings were transcribed and thematically analyzed using a deductive approach that focused on the domains covered in the topic guide (focusing on design, functionality, and content). A generic qualitative approach to thematic analysis was used [61] with interresearcher interpretation. Following familiarization with the transcripts, a member of the team charted themes in a matrix. Possible enhancements and amendments to the toolkit (logged by the developer on GitHub) and field notes taken during the think-aloud sessions were also considered in the analysis process. Subsequently, another team member familiarized themselves with the transcripts and the matrix of initial themes. They developed an agreed coding scheme using an analytical framework that combined a priori issues from the original topic guide and emerging themes [62].

## Results

### Participant Characteristics

Focus groups comprised 8 females and 1 male; all participants had previously attended the face-to-face FACETS program. Self-reported descriptives for the usability testing participants (n=11) are presented in Table 3. All study participants consented to being recorded.

**Table 3:** Self-reported descriptives for usability testing participants (phase 2; n=11).

Characteristic			Value
**Gender, n (%)**			
	Male		4 (36)
	Female		7 (64)
Age (years), mean (SD), range			49 (8.41) 34-62
**Type of MS^a^, n (%)**			
	Relapsing remitting		8 (73)
	Secondary progressive		1 (9)
	Primary progressive		2 (18)
**APDDS^b^, mean (SD), range**			6.8 (2.5) 2-9^c^
	1: mild symptoms that don’t limit activity		—^d^
	2: noticeable symptoms with mild, small impact		2 (18)
	3: limitations on activities of daily living		—
	4: interferes with walking, can walk 300-500 m		—
	5: can walk 100-200 m but often uses a stick		—
	6: needs a stick or single crutch		—
	7: needs 2 canes or walker to walk 20 m		3 (27)
	8: wheelchair is main form of mobility; can move from wheelchair without help		2 (18)
	9: wheelchair is main form of mobility; help needed to move with wheelchair		2 (18)
**Time since diagnosis (years), n (%)**			
	1-5		3 (27)
	6-10		2 (18)
	11-15		3 (27)
	16-20		2 (18)
	>20		1 (9)
**Employment status, n (%)**			
	Working full time (>30 hours per week)		4 (36)
	Unable to work		3 (27)
	Retired		4 (36)
**Use of mobile apps, n (%)**			
	Never use		1 (9)
	Use a few		4 (36)
	Use a lot		6 (55)
**Has attended face-to-face FACETS^e^** **program, n (%)**			
	Yes		7 (64)
	No		4 (36)
**SUS^f^**			
	Median (range)		75 (37.5-95)
	Mean (SD) [95% CI]		74.3 (16.81) [63.2, 85.6]
	**Sauro-Lewis adjective rating, n (%)**		
		A/A+	5 (45)
		B	1 (9)
		C	2 (18)
		D	2 (18)
		E	1 (9)

^a^MS: multiple sclerosis.

^b^APDDS: Adapted Patient-Determined Disease Steps.

^c^Possible scores on the APDDS scale range from 0-10 corresponding to 11 ordinal levels of functioning. However, 2 participants gave ratings indicating they perceived their functioning to fall between ordinals (1 participant between 7-8 and another participant between 8-9) and for the summary statistics these were scored as 7.5 and 8.5, respectively.

^d^Not applicable.

^e^FACETS: Fatigue: Applying Cognitive Behavioral and Energy Effectiveness Techniques to Lifestyle.

^f^SUS: System Usability Scale.

### Focus Groups

Below we summarize the key toolkit requirements suggested by focus group participants:

- Should be a “tool to help rather than a time-consuming task”
- Suitable for those unfamiliar with FACETS
- Important to include positive aspects
- Include self-monitoring feedback (eg, dashboard and graphs)

Below we summarize preferences for the toolkit expressed by focus group participants:

- App should be freely available at app stores
- Reminder (eg, reminders to take rests) and note functions would be useful
- Would like relaxation module to be included
- Linking with Alexa is a good idea

### System Usability Scale

**Figure 3:**
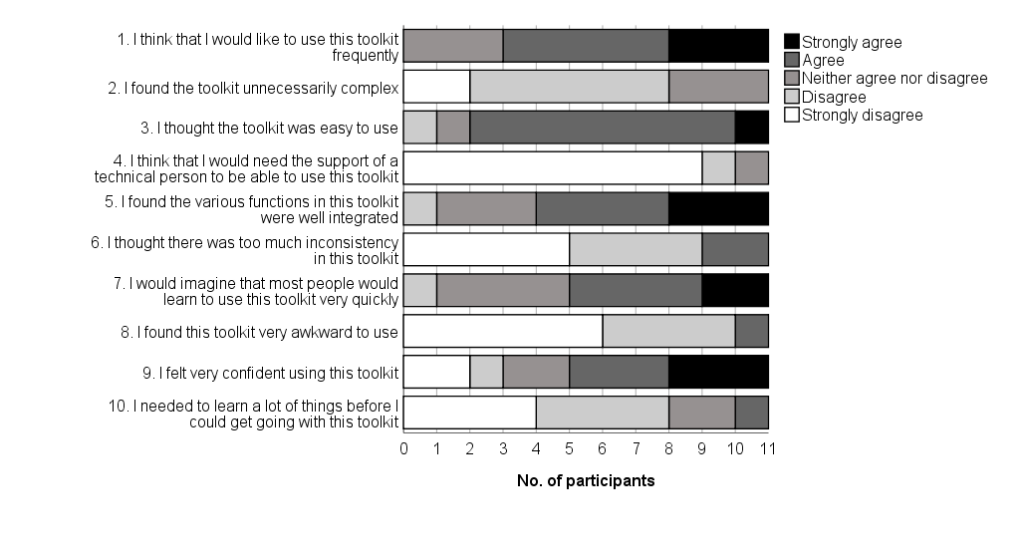
System Usability Scale—item response frequencies.

Summary statistics for the SUS scores are presented in Table 3, and frequencies of ratings for each of the 10 SUS items are presented in Figure 3. Overall, the median SUS score for the toolkit was 75 and the mean was 74.3 (SD 16.81; 95% CI 63.2 to 85.6; range 37.5-95.0). This equates to an adjective rating of good [60] and a B (70th to 79th percentile range) on the Sauro-Lewis curved grading scale [59]. The majority of participants (8/11) thought they “would like to use this toolkit frequently” (the version of the prototype they tested; SUS-Q1). Most considered the toolkit easy to use (9/11; SUS-Q3) with 55% (6/11) believing most people would learn to use it quickly (SUS-Q7). The majority (7/11, 64%) considered the toolkit’s functions to be well integrated (SUS-Q5).

### Usability Feedback

Below we organized the feedback from the usability sessions encompassing general comments and those related to design, functionality, and content, mapped to the toolkit elements. We considered suggestions made for additional functionality. We also included relevant feedback elicited during the focus groups.

#### General Comments

Overall, users found the app relatively easy to navigate, liked its look and feel, and felt that they would quickly get used to the system.

I did like it and I liked the colors because they are bright but they’re not too bright, it was quite, it looked quite vivid I think it is fair to say, which is good. I think it was quite easy to navigate round, I mean once you have got the basic, you know, the three dots and the three lines, from there you can pretty much go anywhere and yes I think it was quite good

P203, Usability sessions

I found my way around easy. I think initially I just had to get to grips with it. Just to look where things were but...I like the menu structure, it was easy to follow.

P204, Usability sessions

However, sometimes users noted difficulty in keeping track of where they were in the app, and early versions of the recap section were found to be confusing.

I’m quite visual and I couldn’t picture the structure of where I was in the...you know...hierarchy of areas, kind of thing, so that was kind of confusing because I then couldn’t then think “Oh I need to skip that bit.”

P202, Usability sessions

Users wanted content and information to be kept brief and easy to understand, without unnecessary repetition and with options to skip content. As one user noted in a focus group in phase 1, it should be “a tool to help rather than a time-consuming task.” Users were in favor of the inclusion of video in addition to audio and text.

You have to be careful not to over...put so much information that it becomes overwhelming, that you can analyze down to so much and you think I can’t think about this anymore and you put too much into it.

P211, Usability sessions

I don’t want to be spending too much time doing it so I’m missing out on doing things...

P205, Usability sessions

Yes you know I am ok reading text but I don’t want to read too much, so you know it is balancing it between video and text.

P207, Usability sessions

Users liked the concept of section-based onboarding and said that prompts and examples were useful, particularly for those with fatigue and cognitive issues. They noted the importance of ensuring reminders are neutral and nonjudgmental in tone.

Yes and you know people with MS we do sometimes get flustered and forget how to do things, so a reminder every now and again, the option to be able to go back and actually see how I do this.

P204, Usability sessions

Yes I think...if somebody is struggling a bit and it keeps saying, “You should be doing this.” But if it said, “Would you like to go?” that, that seems a bit more neutral.

P208, Usability sessions

One user said they would like the app to “look more like a game” (P2011, Usability sessions). The app was seen to be a helpful self-monitoring tool that would be useful for describing fatigue levels, activity patterns, and symptoms to an individual’s clinical team. It was noted that it could also include content that would help family and friends understand more about MS fatigue and its impact.

As well as having the ability to be able to explain to someone who doesn’t have MS, “Do you know what? Watch this video on this app that I’ve got.”

P203, Usability sessions

Users felt that the app could be a useful tool not only alongside the FACETS program but also before or after the program and could be used to gather information prior to a referral by a clinical team for fatigue.

But anyway, it would have been really, really good to have had an app with videos or animations [when newly diagnosed] to talk through the premise of FACETS, almost maybe as a precursor to attending the course.

P203, Usability sessions

Yeah, because my stuff at the moment goes into spreadsheets and when I have to phone up to say, “Oh, I think I am having a relapse,” they ask you a whole bunch of questions: “Have you done this?” “Have you done that?” “When was the last episode you had?” “What is your fatigue like?” All these questions it’s all on a little Excel sheet on my phone at the moment but this will be a bit more of a proper user interface to get that kind of stuff....

P204, Usability sessions

I think for me this is a really hugely useful parallel tool alongside the course or, kind of, after the course

P202, Usability sessions

I mean it would be brilliant for me, I went on the FACETS course a really long time ago, or it feels like a really long time ago now, so to have all of that information would be really good to refresh my memory.

P203, Usability sessions

Users noted that the app could be useful for those with other conditions where fatigue is a symptom.

I think it could be useful...I think it could be infinitely useful for all people with MS, but not just MS, all people who have fatigue, who have a condition that is...causes fatigue.

P208, Usability sessions

#### Activity Diary

##### Feedback

The activity diary was seen to be a helpful way of enabling users to consider activity and fatigue patterns.

Okay, so yes there, that’s quite good for monitoring because actually then you could start to build up a pattern of what makes you feel really fatigued, couldn’t you?

P201, Usability sessions

So it’s as if you can put something in so you don’t overbook yourself you know...and my thing is that I am supposed to have two rest days a week you know, it’s like reminding people it’s fine, rest is part of management really.

P209, Usability sessions

Enabling the user to choose from a prepopulated activity list (and eventually using predictive text suggestions) rather than manual entry was viewed as a useful time-saving feature. It would also make a breakdown of activity type possible.

I think... I think more is probably, I know it’s difficult to put in more, but in terms of fatigue, it’s often the quite nuanced type of activity that is worse or better... it wouldn’t be particularly helpful to have the broad categories because actually work means doing so many different types of things that um...

P202, Usability sessions

The use of location-based services to pull and record relevant information, such as the outside temperature (heat often adversely affects MS fatigue), was suggested by a focus group participant.

P101: ...if it can be linked into the weather, like today it is humid so we have got to think take the shade, keep cool.

Focus Group 1

##### Prototype Changes Made to This Section

Design changes implemented based on usability feedback included implementing swipe to refresh layouts, updating the calendar view originally used to reduce background thread load, fixing a problem whereby activities entered between 12:00 AM and 1:00 AM were not displaying, and revising the layout to improve flow. Location-based suggestions and the addition of predictive text functionality were implemented.

#### Rest and Sleep Planner

##### Feedback

Functionality issues reported included the variable quality of some of the alarm tone sounds included as default on the test phone.

Participant: ...and I wake up to the sound of birds, which is nice, it’s not...

Interviewer 1: Yeah, I was looking at those and they are....they are much more...

Participant: It is nicer than a belting alarm...

P202, Usability sessions

Concern was also expressed by users over the initial layout and legibility of the clock display and rest, wind-up, and wind-down periods chosen (Table 2).

Yeah so if I’m looking at that, right ok, especially with my eyesight problems, I’ve got to read through this whole list to find the thing I want. You’ve got icons next to it, so sleep and wake has got a little cog and half-moon icon next to it but if that...what I mean is if that was bigger and maybe colorful then I could go to it quicker and say right that’s what I want to go back and have a look at my sleep patterns.

P210, Usability sessions

##### Prototype Changes Made to This Section

We amended the visuals to include the duration of wind-up and wind-down periods (Table 2). We also ensured it was easy to read by making the layout tabular in order to separate new rest and sleep/wake routines. Particular attention was paid to customizing how the alarm options could be set and configured. Technical problems that required resolution included issues with the ringtone preview and selection on certain devices, and the alarms not resetting upon phone restart.

#### Goal Planner

##### Feedback

Participants considered the opportunity to set goals a very useful part of the face-to-face program. Suggestions were made to improve user understanding within a screen-based format by offering the ability to view hypothetical worked examples to help convey the concept of SMART (specific, measurable, achievable, and realistic with time for review) goals.

...if you click, 'yes I understand that', then yes it goes on to the next bit or if 'no' then the health care professional would tell you what SMART means because you don’t need another MS person telling you that.

P207, Usability sessions

Users noted that having the ability to select personalized, customizable reminders for entered goals and review and update them could increase engagement with the toolkit.

##### Prototype Changes Made to This Section

The option to note a goal quickly and come back to it later to apply SMART criteria was implemented following user feedback. Case study examples were added to enhance understanding of the concept of SMART goals as this can be difficult for some to understand. Other changes made to improve functionality included adding in pop-up logic to provide guidance to the user when 'no' was selected in the 'realistic' field of the SMART criteria and revising the layout to make it more consistent with the activity diary.

#### Thought Diary and Thought Challenge

##### Feedback

As can be seen in Table 2, elements 4 and 5 of the FACETS homework had been integrated into one section (Thought Diary) to reduce the complexity of screen flow for the user. Feedback from users suggested that although this section was useful, further simplification was required.

Yes I think this thought summary that’s useful because, you had to think about how you were thinking in reflection to the impact it’s had on you, and I found that quite useful to think, because you do exhaust yourself worrying about everything.

P211, Usability sessions

Well, because I’d already rated it once, that was the original frustration of what that incident or whatever was causing me. I don’t think, by the time I’d got to reflecting on the thing and gone back and reviewed what was going on and how I could approach it differently or this, that and the other, I didn’t think it made me any less frustrated or angry from the point that I’d already rated it.

P210, Usability sessions

##### Prototype Changes Made to This Section

The screen flow and structure was simplified further (eg, by removing the requirement to provide ratings for strength of belief in alternative thoughts).

#### Additional Features and Other Issues

Adding a notes function (to enable the quick addition of general pieces of information or thoughts for later expansion or reflection) was suggested as a way of reminding users about historical health-related information (eg, about new symptoms that had emerged, ongoing concerns, other scheduled appointments) that could be useful at MS review consultations (sometimes held a year apart).

When you are newly diagnosed you just, there is this barrage of questions and they don’t come to you when you are sat in front of the consultant or even the nurse and you go home and you think, “Oh god I wish I had asked that.” Oh do you know what I’ve just had a thought, maybe having a notes app, a notes section to write down, you know, notes about your [... ] fatigue or notes about anything else MS-related that you need to talk to your nurse or doctor about, that might be nice.

P203, Usability sessions

I use the notes in my phone a lot, so, er, information that I am told...almost everything, if it doesn’t go into my phone at the time, it gets lost. For instance, I have got the swimming timetable, which I would then put into my fatigue management diary...

P208, Usability sessions

Displaying progress on the dashboard and allowing the user to apply custom queries to data entered (such as their most fatiguing or enjoyable activities) were suggested as ways to enhance engagement. A customizable dashboard could allow the user to create a display containing summary information most relevant and useful to them.

Yes because I would refer back to this and kind of look at what I have done and gauge my fatigue levels.

P204, Usability sessions

It would be good to have one that was a weekly... so you could see what days you were particularly, you know, it’s like actually I need...I have got a lot that I am trying to achieve on that day, so I need to know that I need to have a rest on that day or before....

P207, Usability sessions

Although most participants liked the idea of being able to visualize their fatigue levels over time, one participant felt that this could be disheartening. A customizable dashboard would mean that this feedback could be turned off if a user did not find it helpful.

Yeah if you could turn it on and off or something so you wouldn’t have to see it if you didn’t want to see it or something, yeah it could, if you had that over several weeks and you’re just looking at red, it could be sort of like a bit demoralizing yes.

P205, Usability sessions

Users liked the text-to-speech feature that we included in the recap content.

The fact that you watch or even [with] text you can have somebody saying it at the same time, I find that really helpful for myself, because just reading stuff sometimes it doesn’t, it doesn’t go in properly.

P204, Usability sessions

It helps there was a voice saying the words as well because it breaks it up.

P204, Usability sessions

The text was really easy to read, but having it read to me was really useful.

P208, Usability sessions

Feedback from users in the initial usability sessions suggested they found the synthesized text-to-speech voice overly robotic.

Then as you went on she turned more robotic and more... yes, less human and I think it would be better to be told by somebody sounding a bit more personal. Whether it’s a man or a woman it doesn’t really matter I suppose, it’s just yeah the right person, but yeah.

P205, Usability sessions

As the voice was device-dependent, we only had a certain degree of control over it, but we did find slowing it down a little made it sound more natural.

Usability testers considered the possible integration of voice-activated speakers as a positive method of engagement potentially saving time and energy and requiring less dexterity. In some of the sessions, we gave a demo of inputting a diary entry using Google Assistant, and this was well received.

Yeah. That’s exactly what I would like. Something like that I would use.

P210, Usability sessions

Ensuring that help and support functions on the toolkit accommodated the needs of those unfamiliar with the FACETS program was seen as important. Different types and levels of information would be required for those using the toolkit in conjunction with the FACETS face-to-face program or the e-learning course versus those using it independently. The need for this information to be structured more logically to improve the user journey was highlighted in early usability sessions where users experienced difficulties navigating the recap menu.

So yeah, if I clicked on the activity diary and it says recap, I think I don’t know what that is.

P202, Usability sessions

### Additional Material Incorporated Into Toolkit

We implemented a notes section as suggested, text-to-speech functionality (available in recap sections) was added, and expanded help sections were developed. The concept of the personalized, customizable dashboard was developed gradually as more toolkit elements were introduced and tested over time.

## Discussion

### Principal Findings

In its current form the FACETS toolkit was evaluated as good on the SUS, and qualitative feedback from usability sessions indicated that users felt the toolkit would be useful. Users provided numerous suggestions for improving the toolkit in terms of design, functionality, and content. Some suggestions were implemented immediately following feedback from usability testers. Although the digital format necessitated the simplification of some aspects of the toolkit elements (to reduce cognitive demands and fatigue), it also presented opportunities to create synergies and interactivity between toolkit elements and the dashboard along with visualization and customization possibilities [63].

Findings from previous research suggest that most MS-specific apps lack features desired by people with MS [37,64], resulting in poor uptake [63]. Giunti et al [37] and others have noted the importance of taking into consideration condition-specific factors when designing mHealth apps [39]. The involvement of people with MS throughout the design, prototyping, and usability phases of the toolkit means that such considerations have played a pivotal role. Examples include providing a customizable color scheme (for those with visual difficulties), using icons where possible (to aid memory), providing signposting, and incorporating guidance (eg, buttons to indicate additional scrolled content or the availability of additional information or definitions).

There was some degree of tension between maintaining consistency with the original paper-based FACETS homework tasks and capitalizing on the possibilities afforded by the digital format. The toolkit was developed based on the homework elements of FACETS but did not provide an exact one-to-one mapping. For example, after review of the initial prototype by the team, the thought diary and thought challenge homework elements were combined into one tool as it was felt that the original screen flow was complex to navigate and could lead to frustration or disengagement by users (Table 2) [65].

### Comparison With Prior Work

As noted in the introduction, there are currently few mobile apps for fatigue management in MS. A review of mHealth in MS by Gromisch et al [66] identified 3 mHealth-based apps and 1 web-based platform that promote fatigue self-management through a variety of approaches [66]. These include cognitive behavior therapy principles (MS Energise, which draws upon the FACETS program [30-32]), gamification of energy management via stamina credits (More Stamina [67,68]), and use of validated self-assessments and medication and activity diaries (MSMonitor; web-based platform) [69]. One app (MS Telecoach) focuses on increasing physical activity levels via telemonitoring (accelerometers and self-reported fatigue) and telecoaching (advice, motivational messages, and goal setting) [70].

Similar to findings from the MS Energise [32] and More Stamina [67] usability studies, user feedback for the digital toolkit suggested a need to simplify some aspects. A cross-national qualitative study on facilitators and barriers to using mHealth tools for managing MS highlighted the importance of clear, simple design and features to enhance user accessibility and engagement [40].

Users in the MS Energise usability study suggested text-to-speech functionality would be helpful [32]. We obtained similar feedback in our early usability sessions and incorporated text-to-speech functionality into the recap sections. Users reported finding this useful, describing how it broke up content and aided concentration.

The More Stamina app incorporates gamified elements in the form of stamina credits [67]. In this study, one user said that they would like the digital toolkit to be more game-like. In previous work, we obtained mixed feedback in relation to gamification from both people with MS and health care professionals in the context of fatigue management [12]. Giunti et al [67] found that in their formative work for More Stamina, people with MS reported a preference for collaborative gamified tasks rather than competitive tasks. Untire, a fatigue management app for cancer-related fatigue, incorporates gamified elements (such as progression bars, rewards, and badges) [34]. Gamified elements could be incorporated as optional features in a future version of the digital toolkit. This will be an important area to explore further with users.

The Untire app for cancer-related fatigue allows users to invite a buddy so that they can manage their fatigue with a family member or friend [34]. Feedback from users in this study suggested that the digital toolkit could be a useful tool to help family members and friends understand more about MS fatigue.

In the MS Energise usability study, users felt the app could be particularly helpful for people with MS soon after diagnosis [32]. Similar comments were made in relation to the digital toolkit, and it was seen to offer potential as a tool that could support communication with the clinical team about fatigue and ongoing monitoring, assessment, and treatment. Most testers in this study had been diagnosed for more than 5 years, so further testing of the toolkit is needed with participants who are relatively newly diagnosed.

During field testing of MS Energise, the authors reported that a task involving the identification of unhelpful thoughts in a fictional character gave rise to unhelpful thoughts in some users about their own fatigue [30]. In our study, one user reported that seeing a visual display or heatmap of their own fatigue ratings could be demoralizing. Findings such as these underscore the importance of working closely with users throughout the development lifecycle.

### Prototyping

Due to the power and graphical capabilities of smartphones, mobile apps can be overdesigned [42,71] leading to the paradox of choice [65], where users struggle to process an overwhelming amount of content or too many interface options. The use of online interactive prototyping tools for initial design iterations helped the project team envision their ideas more effectively than with paper alone. They also helped address some of the challenges of working in different locations and across digital and health fields. We recommend the use of an interactive prototyping tool to maximize the advantages of participatory design [72,73] in early design stages. Like Giunti et al [38], we found the use of personas (fictional characters that incorporate composite attributes of target users) a helpful way to capture and convey the varied nature of MS during preliminary stages of development [44].

### Mobile Platforms

A mobile platform offers scope for greater personalization than the paper-based FACETS program materials. As noted, while most participants liked the idea of being able to visualize their fatigue levels over time, one participant noted that this could be disheartening. While self-monitoring can help people with long-term conditions to feel more in control [74], it can also evoke negative emotional reactions [75,76]. In the longer term, we intend to offer customization of the dashboard enabling users to specify the information they wish to visualize or focus on so that it is relevant and meaningful to them. We also explored the best ways to support users to make sense of their self-monitoring data [76].

Suggested methods of highlighting progress included using tracking to document accessed sections and displaying progress on a central dashboard, also highlighted in our previous consultation [12]. Over a longer period of time, this might provide the user (and potentially their health care team) with the ability to track data relating to fatigue and could help to reinforce the FACETS program principles and support users in making lifestyle changes. In the longer term, the toolkit offers possibilities for longitudinal symptom and self-monitoring, data sharing, and greater integration of self-management strategies into daily life, with potential applicability to other long-term conditions. For example, fatigue is a significant and debilitating symptom in a number of neuromuscular disorders [77] and post-COVID-19 [78]. The FACETS program has recently been piloted with a group of long COVID-19 patients using a videoconference delivery format [79].

### Digital Tools

Digital tools like wearables and apps are now starting to help users manage the logistics of their long-term conditions, reminding them to take their medication [80] or helping them manage injection sites [81]. In some conditions like diabetes, significant progress has been made in developing and embedding specific technologies to support self-management [82] and empower patients [22], such as flash glucose monitoring [83]. These have yet to make much impact in MS to date [21-23,64], and concerns over the quality, sustainability, and effectiveness of some solutions remain [84]. Global app stores currently have no direct links with official medical organizations like the NHS or obligations to regulate apps on their behalf. This presents potential risks in terms of inaccurate information being made available and subsequently used within a health context [85,86]. Providing hypothetical worked-through examples within the toolkit elements provided a way of conveying key concepts and illustrating potential benefits from their use. In terms of sustainability, due to the number of devices supporting Android, fragmentation within the current market is huge. Most Android devices are currently using versions of the operating system that are more than 2 years old. Considered targeting of a significant portion of the user base is recommended (rather than trying to target every possible Android legacy device). This is especially important given that new versions of Android and Apple operating systems are released yearly to encourage consumers to upgrade their phones regularly.

The process of developing the digital toolkit was highly iterative and agile; where possible we implemented user suggestions for improvements as we went along. For example, in response to user suggestions, we incorporated the capability for real-time weather data in the activity diary (that could be time stamped at time of input or called back retrospectively) and text-to-speech functionality (available in recap sections).

### Emergent Technologies and Future Enhancements

Emergent technological enhancements (such as Google Voice and Google Assistant) offer further opportunities for improved personalized eHealth solutions [87] and increased engagement [88] and adherence [89]. We are currently exploring the use of voice-activated speakers and assistants such as Google Assistant or Amazon Alexa to enable the input of information via voice rather than keyboard, an innovative way to reduce screen fatigue. We have successfully prototyped this in a closed test with further development planned.

Enhancements could also encompass how a digital toolkit could be used intelligently for smart home-based monitoring and assessment via mobile devices [90]. Emerging technologies such as artificial intelligence, machine learning, and remote monitoring of condition markers could provide opportunities for more tailored and personalized care, from services to treatments [21]. For example, personalized advice and support could be offered based on user responses (in the longer term learned by artificial intelligence) to improve engagement.

Areas for further exploration might focus on the use of the toolkit for self-management and monitoring [64] and facilitating communication with health care or other service providers (such as long-term disability benefits assessors [91]) [39,75]. User suggested enhancements made to the prototype included the ability to add notes, which could be used for meetings with health care providers. Other enhancements might include integration of the toolkit with existing data streams. This could include the ability to collect biometric real-time data by using plug-in oximeters and wearable monitoring devices [92] (although improvements in accuracy are required). There is also the possibility of linking recorded data from the toolkit (such as FACETS attendance, activities logged, and goals set) to secure online sources such as the UK MS Register [93]. The toolkit’s database design and security structures allow for this possibility.

### Strengths and Limitations

A limitation of this research is that the prototype toolkit has only been tested to date in a closed, controlled environment with a limited number of potential users (n=11). It has been argued, however, that 10 or more testers is adequate to identify the majority of usability issues [94]. A further limitation is that currently the toolkit is only available on Android.

Strengths of this study include the multidisciplinary project team, involvement of people with a range of MS types and mobility throughout, the use of mixed methods, and our agile approach to development. Our testing protocols were designed to take fatigue-related issues into account including ensuring testing locations were fully accessible, providing taxis (if required) to the usability testing location, providing refreshments, and incorporating rest breaks.

### Future Research

The keeping-on-track planner (section 5 of the toolkit) is currently still under development. The next phase of the study will involve people with MS remotely testing the prototype using their own Android smartphones and providing feedback via an online semistructured questionnaire (telephone interviews with a subsample). This will enable additional comments, ideas, and development glitches to be identified by a wider range of users across a broader range of mobile devices and actioned prior to release. We will next pilot the toolkit in conjunction with the FACETS program (either face-to-face or virtual) and the e-learning course (similar to the real-world usability testing of MindClimb, an app developed to support skills practice alongside group cognitive behavioral therapy for anxiety in adolescents [20]).

To date, involvement of health care professionals as stakeholders in the development process has been limited. We plan to gather feedback about the toolkit from health care professionals in our next phase of testing. Development of an iOS version has begun, with the toolkit currently in a pre-alpha stage, and this development will continue. Future plans include expanding the app to include more content from the face-to-face program to provide further scaffolding for the tools in the toolkit and self-management support. As the 2018 MS Society/Nuffield Trust data and technology report suggests [21], rather than immediately reaching for data and technology solutions, the future should be constructed within an environment that enables the potential of data and technology to give people with MS the best care possible.

### Conclusions

We have described a mixed methods approach to the design, prototyping, and usability testing of a digital toolkit comprising the homework tasks of FACETS. This work highlights the importance of the participation of people with MS in the development cycle, working to a human-centered design methodology. Continued horizon scanning for emergent technological enhancements will enable us to identify opportunities for further improvements prior to launch.

## Appendix

Multimedia Appendix 1 User requirements topic guide (phase 1).

Multimedia Appendix 2 Interview topic guide (phase 2).

## Abbreviations

FACETS: Fatigue: Applying Cognitive Behavioral and Energy Effectiveness Techniques to Lifestyle
mHealth: mobile health
MoSCoW: must have, should have, could have, and won’t have
MS: multiple sclerosis
RCT: randomized controlled trial
SMART: specific, measurable, achievable, and realistic with time for review
SUS: System Usability Scale
