# Supplementary material for: Creating a Digital Toolkit to Reduce Fatigue and Promote Quality of Life in Multiple Sclerosis: Participatory Design and Usability Study
Source: JMIR Form Res. 2021 Dec 9;5(12):e19230. doi: 10.2196/19230 (PMC8704114; doi:10.2196/19230)
Supplement: Multimedia Appendix 1 [file formative_v5i12e19230_app1.docx]

**Phase 1 Focus Group Topic Guide: User Requirements**

**Creating a FACETS digital toolkit to promote quality of life of people with multiple sclerosis**

*Grand tour question is intended as a broad opening enquiry
The mini tour questions are more focused in nature and will explore issues in greater detail.*

**Take consent / going through the consent form**Explain that the aim is to develop a digital toolkit to support people with MS to monitor and manage aspects of their fatigue.

**Grand Tour Question**

- Which digital devices do you have (e.g. Smartphone, tablet, computer)?

**Mini Tour Questions (use of digital devices) [10 mins]**

- What are some of the positive experiences you’ve had using technology?
- What are some of the negative experiences you’ve had using technology?
- Can you recall times when you’ve used technology to support your MS?
- Do you think that using technology in some way associated with your condition could benefit you?
- Does technology affect you in a negative way or has it had an adverse effect on your condition?
- Have you used online discussion groups or social networks (like Facebook) to talk about your condition or treatment with others?
- How do you use digital devices during your day?

**Mini Tour Questions (use of apps) [10 mins]**

- How do you use apps (if at all) in your-day-to-day life?
- Can you describe some features you liked most on apps used to date?
- Can you describe some features you liked least on apps used to date?
- Have you used any health apps on your mobile phone for information or to support management of your condition?

**Mini Tour Questions (Current FACETS) [10 mins]**

- How long ago did you attend FACETS?
- How has FACETS impacted on your life and your management of fatigue since you completed the programme?
- What were the most useful parts of FACETS to you?
- If you could choose one particular part of FACETS to use on a mobile device what would it be?

*[Prompts: Positive Comments/Changing views on MS, Communication/Group dynamic of FACETS]*

**Mini Tour Questions (mFACETS – Engagement/Homework) [15 mins]**

- Each week, FACETS incorporates ‘homework’ activities - how would you see homework tasks being incorporated into an app?
- How could we encourage people to complete the homework?
- Which homework items did you find most useful?
- Which homework items would you most like to see be digitised and why?
- Would you like the ability to personalise your look and feel?
- Would reminders be useful?
- Would a dashboard or progress bar be useful?
- What would you call this section instead of homework?
- Would you like to store homework information (such as SMART goals) and view it later on?
- Which homework items would you be most likely to use long-term on an app?

*[Prompts: Keeping People Engaged, Keeping in Touch/Reminders, Introducing CBM, Rewards/Goal Setting, Progress Bar/Dashboard]*

----------------- REFRESHMENT BREAK (Look at screen shots) ----------------

**Mini Tour Questions (mFACETS - Design) [15 mins]**

- What are your thoughts about the screenshots you’ve seen?
- How would you like the homework content displayed?
- What device would you be most likely to use a toolkit on for reading; for storing data; for reviewing data?
- Would you like to store information on your device?
- Would you like to access the same information on multiple devices?
- Would you like the option to print or export the information elsewhere?
- What parts of the toolkit would be useful for family and friends to look at (based on them coming along to the first FACETS session)?

*[Prompts: Audience Demographic, Timing and pacing considerations, Look or structural considerations, Online design vs usual, Access to other family members]*

**Mini Tour Questions (mFACETS - Group) [5 mins]**

- Would you be happy talking to others via an app?
- Do you think you’d need access to a facilitator if you were using toolkit content after the programme had finished?
- Would you be worried about storing content of a personal nature on your phone or tablet?

*[Prompts: Group aspect, Trust and safeguarding, Booster Sessions]*

**Mini Tour Questions (mFACETS - Interactivity) [10 mins]**

- Can you suggest any examples of interactive content not relating to MS that you’ve enjoyed?
- Can you suggest any examples of interactive content relating to MS that you’ve enjoyed?
- Would you prefer to read, hear or watch interactive content (e.g. relaxation exercises)?
- Would you prefer to see real people or cartoons in videos?
- Would you prefer to see pwMS or HCPS presenting content?

*[Prompts: Flipcharts, Technical Interactivity, Avatar or real]*

**Mini Tour Questions (mFACETS – HCP Relationships) [10 mins – 12.00]**

- How would you see the toolkit complementing or supporting your care immediately and in the future?

*[Prompts: HCP Involvement, Complementing care]*

**Finally [5 mins]**

- Any other points we haven’t covered or comments you’d like to make?
- Are you happy for us to follow up points of clarification with you via email?

----------------------------------- LUNCH --------------------------------------------------
