# Supplementary material for: Creating a Digital Toolkit to Reduce Fatigue and Promote Quality of Life in Multiple Sclerosis: Participatory Design and Usability Study
Source: JMIR Form Res. 2021 Dec 9;5(12):e19230. doi: 10.2196/19230 (PMC8704114; doi:10.2196/19230)
Supplement: Multimedia Appendix 2 [file formative_v5i12e19230_app2.docx]

| **INTERVIEW** |
| --- |

**Grand Tour Questions**

- What was your first impression of the toolkit? Did you find it easy to use?
- What things did you particularly like?
- What things did you not like about it?
- Was there anything that didn’t work or didn’t work as expected?
- Were there any things about the toolkit that you found frustrating/annoying?
- Did you find it tiring using the toolkit? If yes, are there any changes that could be made to make it less tiring?

**Mini Tour Questions (Design)**

- What did you think about the look of the toolkit?
  - *Prompt: colour*
- Did you find the text easy to read
  - *Prompt: size and colour*
- What did you think about the pictures in the toolkit?
  - *Prompt: did they reflect the information given?*
- Did the toolkit have any annoying features?

**Mini Tour Questions (Usability)**

- Were you able to find your way around the toolkit?
  - *Prompt: Were you able to find what you were looking for?*
- What did you think about the layout of the toolkit?
  - *Prompt: Was it logical?*
- Was it easy to keep track of where you were in the toolkit or did you find yourself getting lost?

**Mini Tour Questions (Information Quality)**

- In terms of the information in the toolkit– was there too much content, not enough or about right?
- In terms of the explanations provided in the toolkit - were there too many, too few or about the right amount?
- The toolkit contains the FACETS homework tasks – do you think any key information was missed out?* [**only for people who have attended FACETS*)

**Mini Tour Questions (Version Specific examples)**

- What are your thoughts on the tutorial?
- What did you think about the recap section for the activity diary?
- Were you able to add an activity diary entry?
  - *Prompt: How did you find the process of rating fatigue and enjoyment levels of the activities you entered?*
  - *Prompt: Were the instructions easy to follow/intuitive?*
  - *Prompt: Were you able to review your activities?*
  - *Prompt: If the toolkit provided lists of activities for you to choose rather than enter manually do you have any preferences for how these would be displayed?*
- Would you like the option of selecting different activity categories rather than entering them manually? If so, how would you like to enter them?
  - *Prompt: by category and sub-category*
- Do you have any ideas on how we could display activity information on a heatmap? (a representation of data where values contained in a picture are represented by colours)
  - *Prompt: Show picture of heatmap graphic*
- What did you think about the recap section for the rest/sleep/wake planner?
- How did you find the rest/sleep/wake planner?
  - *Prompt: Were you able to add an alarm/notification*
- Were you able to add a goal successfully?
  - *Prompt: How did you find the process of entering SMART information related to your goal?*
  - *Prompt: Were the instructions easy to follow/intuitive?*
  - *Prompt: Were you able to review your goals OK?*
- Were you able to add a situation, unhelpful thoughts and emotions successfully?
  - *Prompt: Were the instructions for this section clear and intuitive?*
  - *Prompt: How did you find the process of challenging your situation?*
  - *Prompt: Were you able to select and deselect thinking styles easily?*
  - *Prompt: Were you able to reflect and re-rate your thoughts and emotions OK?*
  - *Prompt: Do you think this section is too complicated/long for a user to complete?*
- Do you have any suggestions for improvement, based on what you have seen so far?
- Are there other features you would you like to see in this toolkit?
- From what you have seen so far, would you like to test a later version of the toolkit?

**Mini Tour Questions (Wider Toolkit Use)**

- Do you think other people with MS would find this toolkit helpful?
- Do you think that this toolkit would help people with different types of MS, at any stage of development?
  - Prompt: Do you think it would be particularly useful to those that have been recently diagnosed and/or those that have had MS for some time?
